# Identification of CD36 as a contributor in inflammatory response of rheumatoid arthritis and screening of feasible bioactive drugs targeting it

**DOI:** 10.1186/s41065-025-00450-3

**Published:** 2025-06-03

**Authors:** Dan Xuan, Xiaowan Wang, Dandan Feng, Li Wang, Yonghui Xia

**Affiliations:** 1 Department of Rheumatology and Immunology, The First Affiliated Hospital of Wannan Medical College, Wuhu , Anhui, 241000 China; 2 Department of Respiratory and Critical Care Medicine, The Second Affiliated Hospital of Wannan Medical College, Wuhu , Anhui, 241000 China

**Keywords:** Rheumatoid arthritis, WGCNA, Virtual screening, CD36, Inflammatory response, Fibroblast-like synovial cells

## Abstract

**Background:**

Rheumatoid arthritis (RA) is a chronic inflammatory disease. This study aims to identify candidate therapeutic targets and promising drugs for RA.

**Methods:**

RA-related microarray datasets (GSE77298 and GSE206848) and inflammatory genes (IRGs) were downloaded from Gene Expression Omnibus database and GeneCards database, respectively. After removing batch effects, differentially expressed genes (DEGs) were screened using filtering criteria of *P* < 0.05 and |log2(fold change)|> 1. Differentially expressed IRGs (DE-IRGs) were then obtained. Key gene modules were identified by weighted gene co-expression network analysis (WGCNA), and the hub genes were then identified from the results of protein–protein interaction (PPI) network analysis, WGCNA and DE-IRGs, and validated by a external dataset GSE93272. Receiver operating characteristic (ROC) curve was used to evaluate the diagnostic effect of the predicted hub genes. In addition, drug prediction was performed through virtual screening. mRNA and protein expression of cluster of differentiation 36 (CD36) were detected by RT-qPCR and Western blot. After RA fibroblast-like synovial cells (RA-FLS) were treated with piceatannol and epicatechin, cell proliferation was detected by CCK-8 assay, and flow cytometry was used to detect cell cycle and apoptosis, and the secretion of inflammatory cytokines was detected by enzyme-linked immunosorbent assay.

**Results:**

Three hub genes were finally identified, including CD36, perilipin 1 and lipoprotein lipase. CD36 was further identified as a candidate biomarker and therapeutic target for RA, which had relatively good diagnostic efficacy for RA. Compared with fibroblast-like synovial cells (FLS), mRNA and protein expression levels of CD36 in RA-FLS were significantly up-regulated (*P* < 0.05). Piceatannol and epicatechin had good binding affinity with CD36 (docking score < -5 kcal/mol), and piceatannol treatment or epicatechin treatment inhibited the proliferation and inflammation of RA-FLS and induced cell cycle arrest and apoptosis (*P* < 0.05).

**Conclusion:**

CD36 is a potential biomarker and therapeutic target associated with synovial inflammation of RA, and piceatannol and epicatechin are potential natural drugs for RA treatment. Overall, these findings provide new insights into the clinical diagnosis and treatment of RA.

## Introduction

Rheumatoid arthritis (RA) is a chronic inflammatory autoimmune disease characterized by synovial hyperplasia, inflammatory response, and cartilage and bone destruction [1]. Currently, the overall global prevalence of RA is 0.46%[2], and women are at a higher risk of developing the disease, two to three times that of men [3]. RA can occur at any age and is most common between the ages of 40 and 60 [4]. If left untreated, RA may develop small focal necrosis, granulation adhesion, and fibrous tissue on the surface of the joint, leading to progressive joint stiffness, destruction, deformity, and disability [5]. In addition, about 40% of RA patients have complications, and the existence of complications seriously reduces the quality of life of RA patients and even leads to an increase in mortality [6]. Despite significant advances in treatments, many patients with RA do not respond effectively to current treatments, and additionally the current treatment strategies may cause serious side effects [7]. Therefore, it is necessary to clarify the mechanism of RA pathogenesis and find new strategies to treat RA.

Genetic and environmental factors contribute to the pathogenesis of RA [8]. Fibroblast-like synovial cells (FLS) are important components of synovium of joint, and maintain joint function by producing extracellular matrix (ECM) and synovial fluids [9]. However, under inflammatory conditions such as RA, the phenotypes of FLS are changed, and the proliferation and invasiveness of FLS are promoted, facilitating RA pathogenesis and aggravating clinical symptoms [10, 11]. Rheumatoid arthritis FLS (RA-FLS) have been reported to have similar properties to tumor cells, such as excessive proliferation, apoptotic resistance, increased invasiveness, and production of inflammatory mediators [12]. Excessive proliferation of RA-FLS leads to synovial inflammation and cartilage degradation [13]. Therefore, targeting RA-FLS is a potential strategy for RA treatment.

**Fig. 1:**
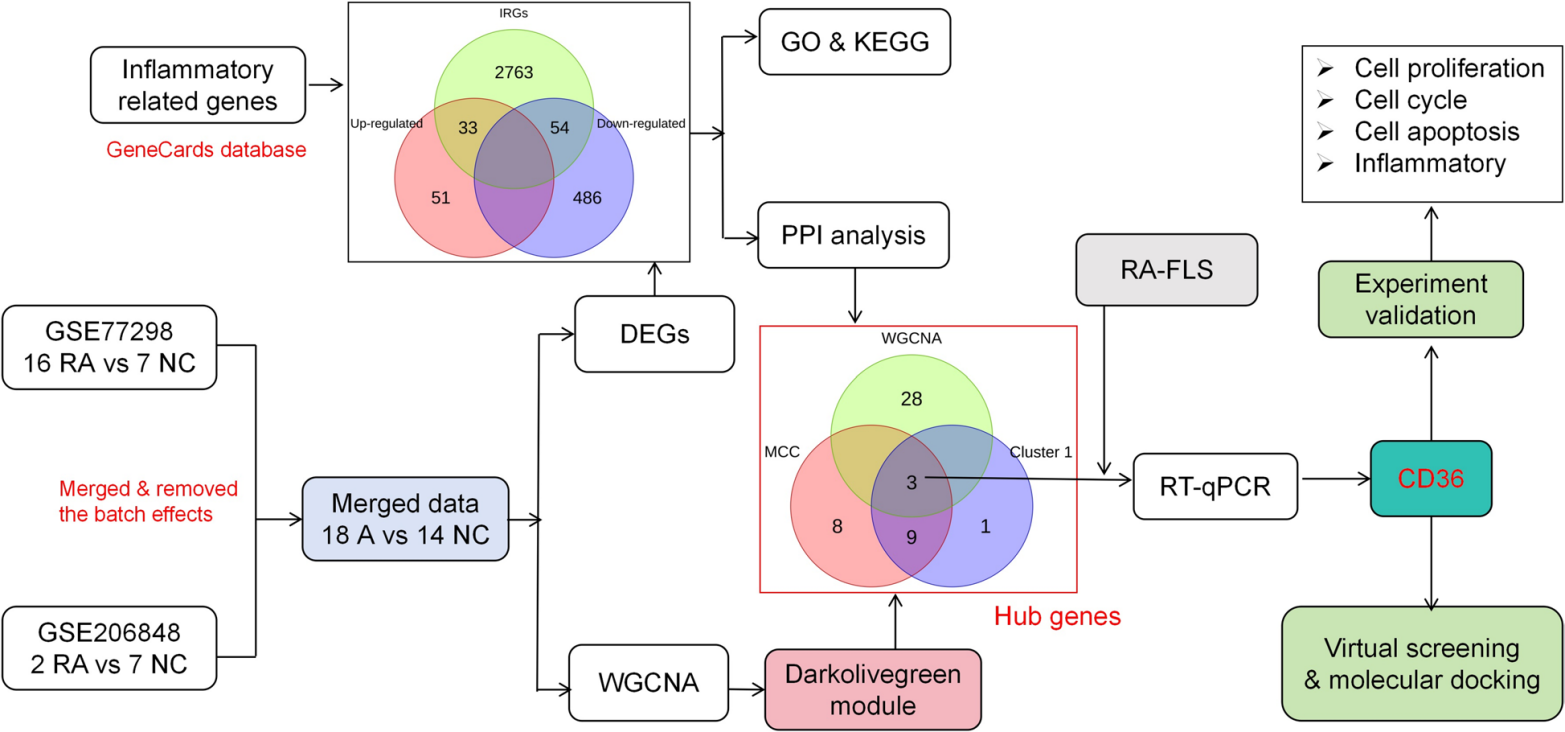
Flowchart of this study

This study aimed to explore potential biomarkers and therapeutic targets in RA. Firstly, differentially expressed genes (DEGs) in synovial tissue of RA were identified, and then hub genes associated with RA progression were then obtained using weighted gene co-expression network analysis (WGCNA) and protein–protein interaction (PPI) network analysis. In addition, natural drugs targeting the hub target cluster of differentiation 36 (CD36) were explored using virtual screening. Finally, the regulatory effects of piceatannol and epicatechin on the phenotypes of RA-FLS were investigated by in vitro models. The flowchart of this study is summarized in Fig. 1.

## Methods and Materials

### Data download and processing

RA-related datasets were searched in the Gene Expression Omnibus (GEO) database (http://www.ncbi.nlm.nih.gov/geo)."Rheumatoid arthritis"was used as the key search term. The “Study type” was defined as “expression profiling by array”, and the “platform” was defined as “GPL570”, and the “Organisms” was defined as “*Homo sapiens*”. Sixty-three datasets were retrieved, and the datasets focusing on certain treatments on gene expression profile of RA tissues were excluded, and the datasets focusing on comparing gene expression profile between RA patients and healthy controls were included. Finally, three microarray datasets (GSE77298, GSE206848 and GSE93272) were obtained. GSE77298 dataset contains the gene expression profile data of 23 synovial tissue samples from 16 RA patients and 7 healthy individuals. GSE206848 dataset contains the gene expression profile data of synovial tissue samples from 2 RA patients and 7 healthy controls. The GSE93272 dataset contains the gene expression profile data of peripheral blood samples from 232 patients with RA and 43 healthy volunteers. R software package inSilicoMerging [14] was used to merge GSE77298 and GSE206848 datasets. The ComBat algorithm was used to remove batch effects, and the normalizeBetweenArrays algorithm was used to normalize gene expression in different samples. The batch-effect removal was assessed using box plots of gene expression profile and Uniform Manifold Approximation and Projection (UMAP).

### Identification of differentially expressed inflammation-related genes (DE-IRGs)

The combined dataset was analyzed using the R package limma (version 3.40.6) to obtain differentially expressed genes (DEGs) in synovial tissue samples between the normal controls (NC) and the patients with RA. All P-values were False Discovery Rate (FDR)-corrected using the R function P.adjust. However the number of the obtained DEGs was insufficient for the subsequent analysis. So finally, the screening criteria for DEGs were defined as “*P* < 0.05” and “|log_2_fold change|> 1”. In addition, inflammation-related genes (IRGs) were obtained from GeneCards database with"inflammation"as the key word. Relevance score ≥ 1.35 (mean) was used as screening criterion. Collectively, a Venn diagram was used for obtaining the DE-IRGs. Gene ontology (GO) analysis and Kyoto Encyclopedia of Genes and Genomes (KEGG) pathway enrichment analysis of DE-IRGs were performed using the R package clusterProfiler. *P* < 0.05 was considered as the threshold.

### WGCNA

The gene co-expression network was constructed using the"WGCNA"package in R software. First, softConnectivity and pickSoftThreshold were used to determine the soft threshold power (β) for building a scale-free network. Then, topological overlap matrix (TOM) was used to construct network interconnectedness, and gene modules were identified based on hierarchical clustering method. In addition, the correlation between the module and RA was calculated based on the characteristic genes. The module with the strongest correlation with RA was selected as the key module for subsequent analysis. Genes with |module membership (MM)|> 0.8 and |gene significance (GS)|> 0.1 were selected as candidate genes for key modules.

### Construction and analysis of PPI network

DE-IRGs were imported into STRING database (https://cn.string-db.org/) for PPI network construction. The species was set as “*homo sapiens*”, and the minimum interaction score required was set as “medium confidence” (0.4). After removing the free nodes, the PPI network map was obtained, and the TSV file was downloaded and saved. The PPI network was then evaluated using the CytoHubba plug-in based on the maximal clique centrality (MCC) algorithm. The top 20 genes were selected as candidate genes. In addition, the molecular complex detection (MCODE) plug-in in Cytoscape 3.9.0 software was used to identify key gene clusters in the PPI network according to the default parameters. The default parameters were: degree cutoff = 2, node score cutoff = 0.2, K-score = 2, and max depth = 100.

### Identification and validation of hub targets

Using BioLadder online platform (https://www.bioladder.cn/web/#/chart/17), the hub targets of RA pathogenesis were screened out from the results of WGCNA, MCC algorithm and MCODE plug-in analysis, based on a Venn diagram. In addition, based on GSE93272 dataset, R package ggpubr was used to generate the expression box plots of the hub targets. The receiver operating characteristic (ROC) curve was plotted using the pROC package in R language, and the area under the ROC curve (AUC) values were calculated, and the ROC curve was visualized using the ggplot2 package.

### Virtual screening and molecular docking

A natural product library was used for this virtual screening. The molecular structure of the CD36 protein was obtained from RCSB Protein Data Bank database (https://www.rcsb.org/), and the structure of natural products were obtained from PubChem Compound database (https://www.ncbi.nlm.nih.gov/pccompound/?term =). The Prep Wiz module in Schrödinger software was used for processing CD36 protein with hydrogenation and water removal. All of the natural monomer were preliminarily screened for using Lipinski's rule of five and Veber rule modules in DS4.0. The remaining natural monomers were prepared with the LigPrep module in the Schrödinger software. OPLS_2005 force field was used, and the protonation of the molecule was conducted under pH7.4 conditions using the Epik module. The SiteMap module of Schrödinger software package was used to predict the active pocket of CD36 protein. The optimal docking condition Glide field was selected and the CD36 protein file was used as the receptor for docking analysis. The active pocket of the protein receptor was defined as a pocket with the size of 10 Å × 10 Å × 10 Å. Glide algorithm was used for docking with standard docking accuracy, and the other parameters remained default. The natural monomers were ranked by docking scores.

### Cell culture

Human normal FLS and RA-FLS were purchased from Zeye Biotechnology Co., Ltd. (Shanghai, China). The cells were cultured in Dulbecco's Modified Eagle Medium/Nutrient Mixture F-12 (DMEM/F12; Gibco, Grand Island, NY, USA) supplemented with 10% fetal bovine serum (FBS; Gibco, Grand Island, NY, USA) and 1% penicillin/streptomycin (Gibco, Grand Island, NY), in a humidified incubator containing 5% CO_2_ at 37 °C. Piceatannol, epicatechin and methotrexate (MTX) (Beyotime, Shanghai, China) were dissolved in dimethyl sulfoxide (Beyotime, Shanghai, China), and diluted by the medium to treat the cells, respectively.

### Cell proliferation assay

Cell counting kit-8 (CCK-8) was used to detect the proliferation of RA-FLS. RA-FLS was inoculated into 96-well plates at a density of 5000 cells/well and cultured overnight at 37℃. 10 μl CCK-8 solution was added to each well at the specified time point (0, 24, 48, 72 or 96 h) and incubated at 37℃ for 2 h. The absorbance (optical density, OD) values of each well at 450 nm were detected by an automatic enzyme-labeling instrument (Bio-Rad, Hercules, CA, USA).

### Reverse transcription-quantitative polymerase chain reaction (RT-qPCR)

Total RNA was extracted from normal FLS and RA-FLS by TRIzol reagent (Invitrogen, Carlsbad, CA, USA). Total RNA was reverse-transcribed into complementary DNA (cDNA) with A PrimeScript RT Reagent Kit (Takara, Dalian, China). According to manufacturer's instructions, a SYBR Premix Ex Taq II kit (Takara, Dalian, China) was used for cDNA amplification in 7500 real-time PCR instrument (Applied Biosystems, Foster City, CA, USA). Glyceraldehyde-3-phosphate dehydrogenase (GAPDH) was used as the internal reference gene. The sequence of primers used were as follows: CD36 forward 5'-GTTGAGAGCCTGTGCCTCAT-3'and reverse 5'-TGCAGGAGCAGATGCAGAA-3'; perilipin 1 (PLIN1) forward 5'-CGGTCAGCCGGACTTGAG-3'and reverse 5'-GGGTGGAGATGGTGTCCTTC-3'; lipoprotein lipase (LPL) forward 5'-AGTAGCAGAGTCCGTGGCTA-3'and reverse 5'-GGGACCCTCTGGTGAATGTG-3'; GAPDH forward 5'-GTCTCCTCTGACTTCAACAGCG-3'and reverse 5'-ACCACCCTGTTGCTGTAGCCAA-3'.

### Western blot assay

Total protein was extracted from normal FLS and RA-FLS using RIPA lysis buffer (Beyotime, Shanghai, China) containing protease inhibitors (Roche, Mannheim, Germany). An equivalent amount of protein (20 μg per lane) was separated by sodium dodecyl sulfate–polyacrylamide gel electrophoresis and subsequently transferred to a polyvinylidene fluoride membrane (Millipore, Billerica, MA, USA) with wet method. The membrane was blocked with 5% skim milk at room temperature for 1 h, followed by incubation with primary antibodies: anti-CD36 antibody (ab133625, 1:1000), anti-PLIN1 antibody (ab172907, 1:1000), anti-LPL antibody (ab91606, 1:1000), and anti-GAPDH antibody (ab9485, 1:1000) at 4 °C overnight. The membrane was then incubated with goat anti-rabbit IgG H&L (ab6721, 1:5000) at 37 °C for 2 h. Finally, the protein bands on the membranes were developed using the BeyoECL assay kit (Beyotime, Shanghai, China), and quantitative analysis was performed using Image Lab™ software (Bio-Rad, Hercules, CA, USA). All antibodies used in this study were purchased from Abcam (Shanghai, China).

### Flow cytometry

RA-FLS was trypsinized and resuspended, and single-cell suspension was prepared. Then the RA-FLS were fixed overnight with 75% ethanol at 4 °C. The fixed cells were washed and stained with 25 mg/ml propidium iodide (PI; Beyotime, Shanghai, China) containing 0.1% Triton X-100 and 10 mg/ml RNase in the ice for 30 min in the dark. After the cells were washed with phosphate buffer saline (PBS), the cell cycle distribution was analyzed on the FACSCalibur system (BD Biosciences, San Jose, CA, USA). The percentage of RA-FLS in different cell cycle stages was calculated using ModFit 3.0 (Verity Software House, Inc., Topsham, ME, USA). An Annexin V-Fluorescein Isothiocyanate (FITC)/PI apoptosis detection kit (Beyotime, Shanghai, China) was used to detect the apoptosis of RA-FLS. The cells were washed twice with cold PBS. The RA-FLS were re-suspended in 100 μL binding buffer containing 10 μL Annexin V-FITC and 10 μL PI, and stained at room temperature in the dark for 30 min. After washing with PBS, the stained cells were analyzed within 1 h, and the rate of apoptosis was analyzed using FlowJo V.10 software.

### Enzyme-linked immunosorbent assay (ELISA)

RA-FLS was inoculated into 6-well plates at a density of 4 × 10^3^ cells/well and cultured overnight. The cells were then stimulated with 10 ng/mL tumor necrosis factor-α (TNF-α; Beyotime, Shanghai, China) for 24 h, and then treated with piceatannol and epicatechin. Next, the cells were centrifuged at 2000 × g at 4℃ for 10 min and the supernatant was obtained. Subsequently, the corresponding human ELISA kits (Beyotime, Shanghai, China) were used to detect the concentrations of inflammatory cytokines interleukin-6 (IL-6), interleukin-1β (IL-1β) and interleukin-8 (IL-8) in the supernatants according to the manufacturer’s instructions.

### Statistical analysis

All experiments were conducted independently in triplicate. All data are expressed as mean ± standard deviation (SD). SPSS 21.0 software (IBM Corp., Armonk, NY, USA) was used for statistical analysis, and *P* < 0.05 indicated significant statistical difference.

## Results

### Identification by DEGs

**Fig. 2:**
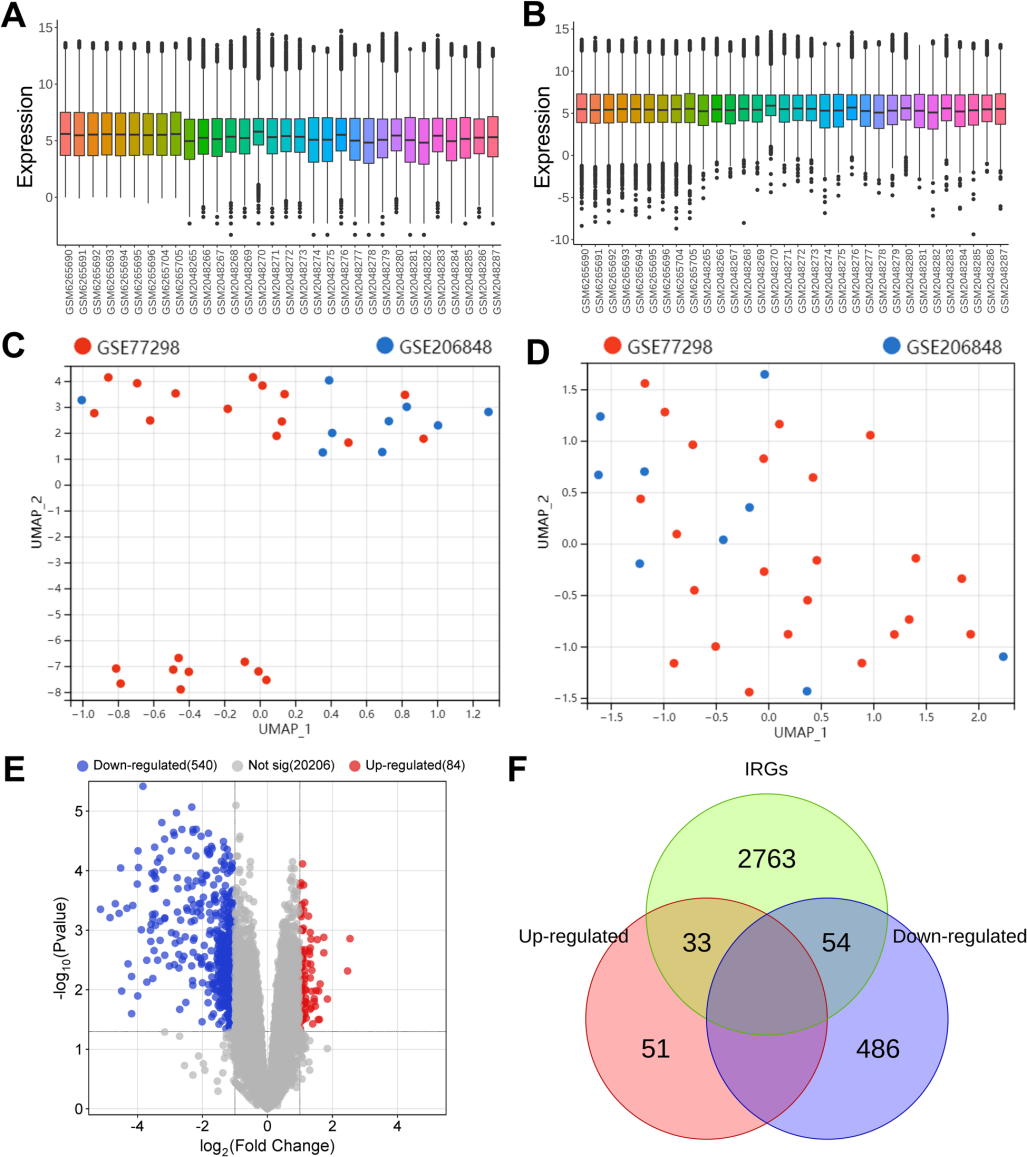
Identification of DEGs in synovial tissue of RA. A&B. Box plots show the sample distribution of each data set before (A) or after (B) removal of batch effects. C&D. UMAP of sample distribution of each dataset before (C) or after (D) removal of batch effect. E. Volcanic maps show DEGs between the RA group and the normal control (NC) group. Red means up-regulated genes, blue means down-regulated genes, and gray means the difference is not significant. F. A Venn diagram is used to obtain DE-IRGs

Firstly, inSilicoMerging was used to merge GSE77298 and GSE206848 datasets, and ComBat was used to remove batch effects. It was observed from the box plots that the sample distribution of the two datasets before batch effect removal was quite different (Fig. 2A); after the removal of the batch effect, the gene expression distribution among all samples from the two datasets was consistent (Fig. 2B). UMAP showed that samples from the two datasets were clustered together before the removal of the batch effect (Fig. 2C), and samples were interwoven after the removal of the batch effect (Fig. 2D). After normalization of the data, differential analyses were performed with threshold parameters *P* < 0.05 and |log_2_ fold change|> 1. A total of 624 DEGs were identified, including 540 down-regulated genes and 84 up-regulated genes (Fig. 2E). In addition, after searching, 2850 IRGs were obtained from the GeneCards database using"inflammation"as a keyword. Subsequently, cross analysis of DEGs and IRGs was performed, and a total of 87 DE-IRGs were obtained (Fig. 2F).

### GO analysis and KEGG pathway enrichment analysis of DE-IRGs

**Fig. 3:**
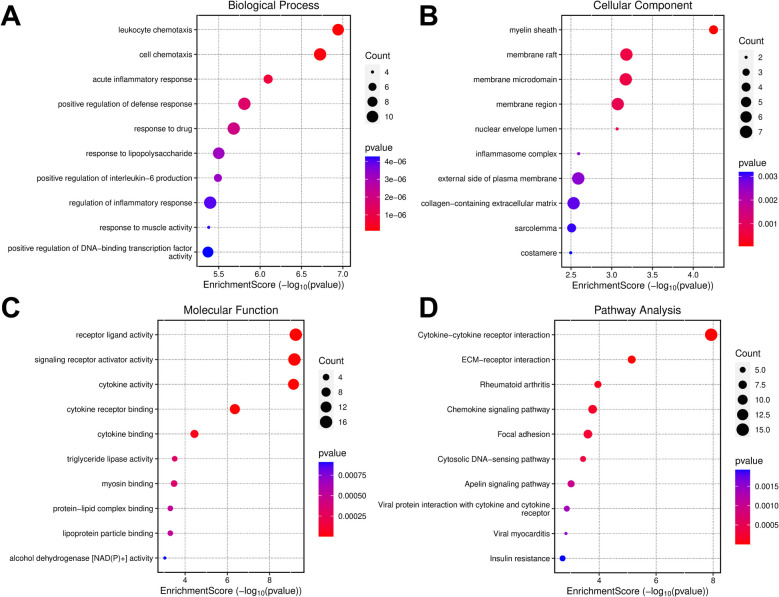
Functional enrichment analysis of DE-IRGs. A-D. Bubble maps show the enrichment analyses results of GO biological process (A), GO cell component (B), GO molecular function (C), and KEGG pathway (D) of DE-IRGs. The bubble size represents count, and the bubble color represents the *p*-value

To elucidate the potential functions and mechanisms of DE-IRGs, GO analysis and KEGG pathway enrichment analysis we. GO analysis showed that DE-IRGs were enriched in 932 GO terms (*P* value < 0.05), including 808 biological processes, 43 cell components and 81 molecular functions. In terms of biological processes, DE-IRGs were mainly associated with leukocyte chemotaxis, cell chemotaxis, acute inflammatory response, and positive regulation of defense response is related to response to drug (Fig. 3A). In terms of cell components, DE-IRGs were mainly enriched in myelin sheath, membrane raft, membrane microdomain, membrane region and nuclear envelope lumen (Fig. 3B). In molecular function, DE-IRGs were mainly involved in receptor ligand activity, signaling receptor activator activity, and cytokine activity and cytokine receptor binding and cytokine binding (Fig. 3C). In addition, the results of KEGG pathway enrichment analysis showed that the DE-IRGs were enriched in 43 pathways (*P* value < 0.05). The top 10 pathways are cytokine-receptor interaction (hsa04060), ECM-receptor interaction (hsa04512), RA (hsa05323) and chemokine signaling pathway (hsa04062), focal adhesion (hsa04510), cytosolic DNA-sensing pathway (hsa04623), apelin signaling pathway (hsa04371), viral protein interaction with cytokine and cytokine receptors (hsa04061), and viral myocarditis (hsa05416) and insulin resistance (hsa04931) (Fig. 3D).

### WGCNA identified the candidate genes involved in RA pathogenesis

**Fig. 4:**
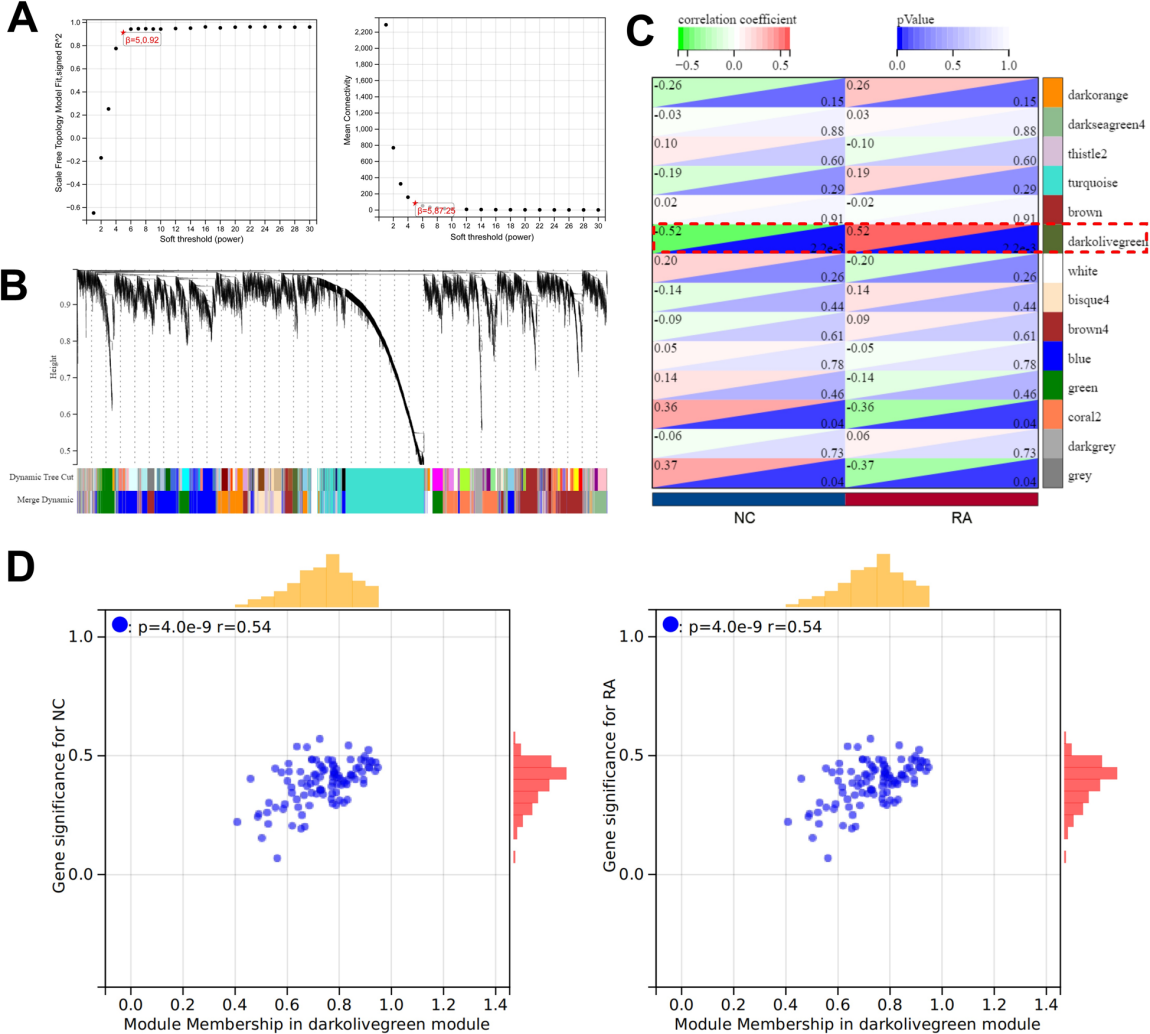
Identifying key RA-related gene modules through WGCNA. A. Scale-free network analysis of different soft threshold power (β). B. Clustering tree of genes. Each branch represents a gene, and each color below represents a co-expression module. C. Heat map shows the correlation between module feature genes and clinical features. D&E. The scatter plots show the correlation between module membership (MM) and gene significance (GS) of the genes in darkolivegreen module in the NC (D) and RA (E) groups

To explore the key gene modules in RA pathogenesis, a gene co-expression network was constructed using WGCNA. A power of β = 5 was set as a soft threshold parameter to construct the scale-free network (Fig. 4A). Furthermore, we calculated the dissimilarity of the module feature genes, selected the tangents of the module tree, and merged the modules with a distance of less than 0.5, and finally obtained 14 co-expression modules (Fig. 4B). Module-feature correlation analysis showed that darkolivegreen module had the largest negative correlation with NC sample and the largest positive correlation with RA sample (Fig. 4C). Furthermore, the correlation plots of MM and GS showed that the genes in the darkolivegreen module were significantly correlated with the NC and RA samples (Fig. 4D). Therefore, the darkolivegreen module, which contained 31 candidate genes, was identified as the key module.

### Identification and validation of hub targets

**Fig. 5:**
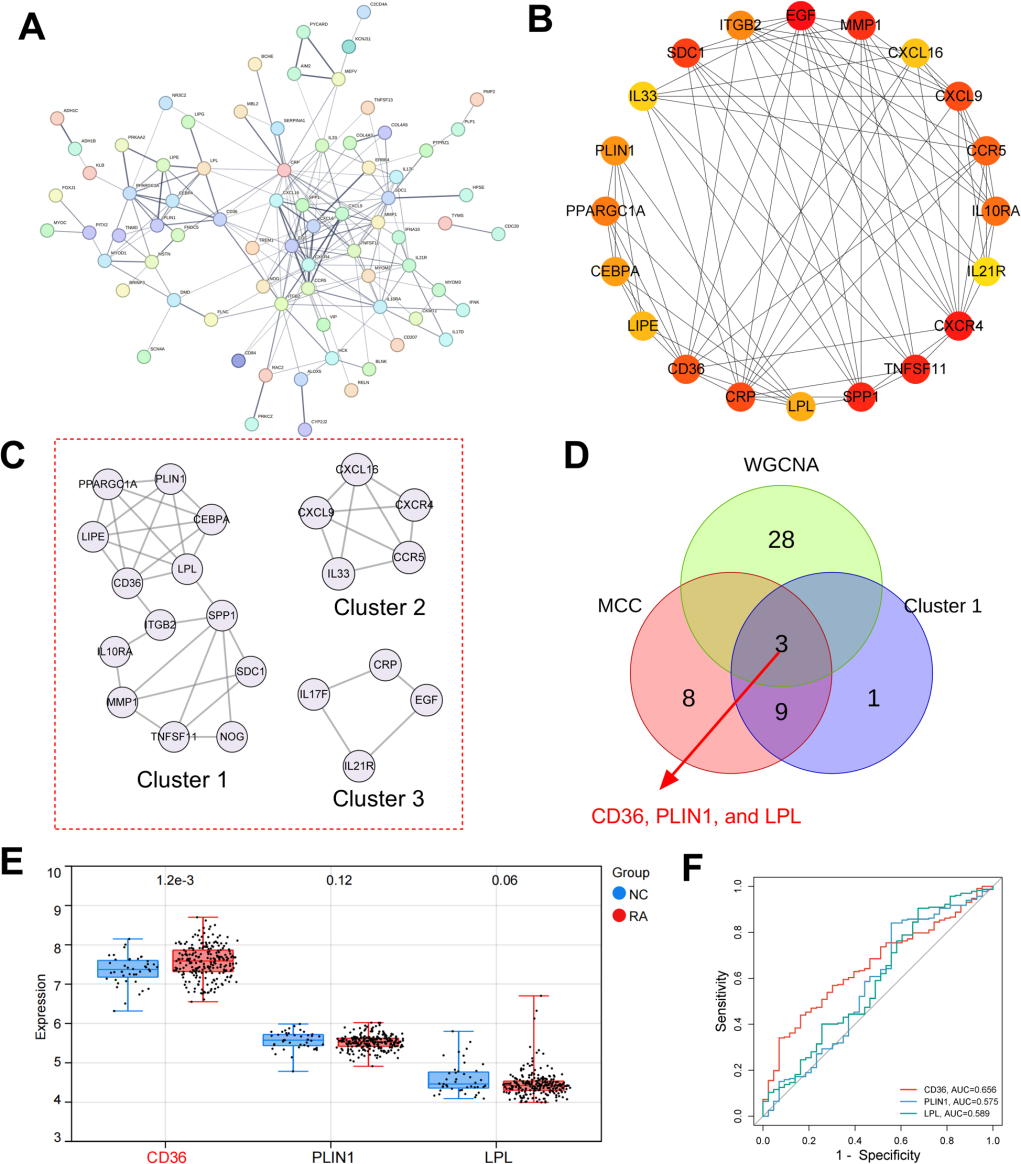
Identification and verification of hub gene. A. STRING database is applied to construct the PPI network based on the DE-IRGs. Nodes represent proteins, and edges represent protein–protein interactions. B. CytoHubba plug-in is applied to extract the top 20 genes in PPI network based on MCC algorithm. C. Cluster analysis of PPI network using MCODE plug-in. D. A Venn diagram is applied to obtain the genes in the intersection of the identified genes from WGCNA (darkolivegreen module), MCC algorithm, and MCODE plug-in (cluster 1). E&F. The expression of CD36, PLIN1 and LPL genes in GSE93272 dataset was analyzed by Willcoxon rank-sum test. Blue represents the normal control (NC) group and red represents the RA group. ROC curves for CD36, PLIN1, and LPL genes in the G&H.GSE93272 dataset

**Fig. 6:**
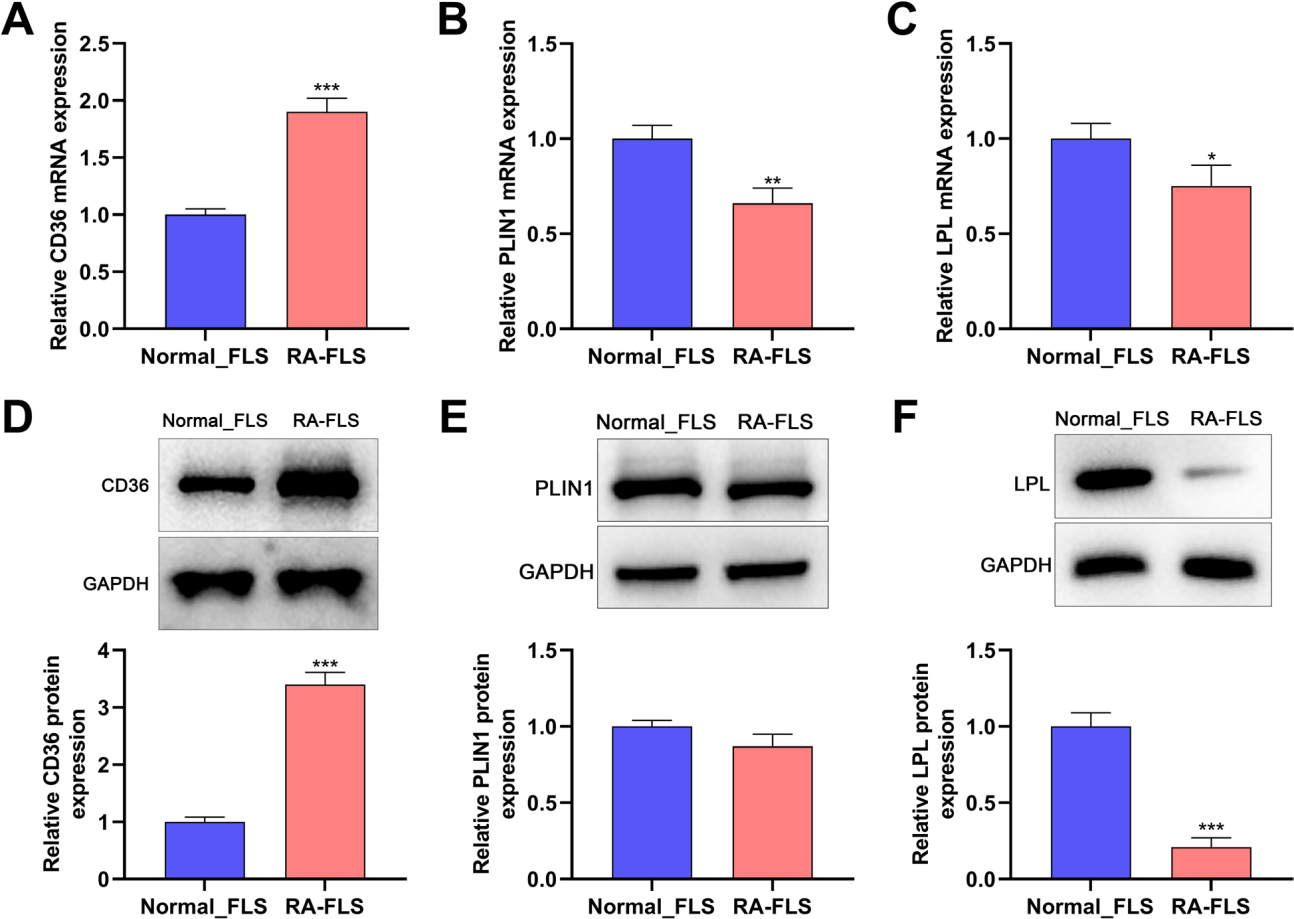
CD36 was highly expressed in RA-FLS. C. qRT-PCR was applied to detect the expression levels of CD36 (A), PLIN1 (B) and LPL (C) in RA-FLS and FLS. D-F. Western blot was applied to detect the expression levels of CD36 (D), PLIN1 (E) and LPL (F) in RA-FLS and FLS. *P < 0.05, **P < 0.01, ***P < 0.001

In order to explore the hub targets of RA, a PPI network of the 87 DE-IRGs was constructed using STRING online platform, and the PPI network consisted of 75 nodes and 186 edges (Fig. 5A). The average node degree of the network was 4.38, and the avg. local clustering coefficient was 0.474. CytoHubba plugin was used to score each node in the PPI network based on the MCC algorithm, and the top 20 genes were identified (Fig. 5B). In addition, a cluster analysis of PPI networks was performed using the MCODE plug-in to generate three highly connected gene clusters (Table 1). Among them, cluster 1 had the highest score, which contained 13 nodes and 28 edges (Fig. 5C). The key genes identified by WGCNA, MCC algorithm, and MCODE plug-in were then cross-analyzed (Fig. 5D). Finally, three hub targets were obtained, including CD36, PLIN1 and LPL. To verify the reliability of the predicted hub targets, we verified their expression based on the GSE93272 dataset. As shown (Fig. 5E), the expression of CD36 in peripheral blood of RA patients was significantly higher than that in NC group, while the expression of PLIN1 and LPL was not significantly different. ROC curve showed that the AUC value of CD36 in the GSE93272 dataset was greater than 0.6 (Fig. 5F), suggesting that CD36 level in blood sample was relatively specific and sensitive for the diagnosis of RA. Therefore, CD36 was identified as a hub biomarker and therapeutic target for RA. To further validate the expression of hub targets in RA, we used RT-qPCR and to detect their expression in normal FLS and RA-FLS. As shown (Figs. 6A-F), compared with normal FLS, CD36 mRNA and protein expression levels were significantly up-regulated in RA-FLS, while mRNA and protein expression levels of PLIN1 and LPL were not changed or significantly down-regulated in RA-FLS.

**Table 1:** Cluster analysis of protein–protein interaction network

Cluster	Score	Nodes	Edges	Node IDs
1	4.667	13	28	TNFSF11, MMP1, IL10RA, PPARGC1 A, CD36, LPL, CEBPA, LIPE, SPP1, ITGB2, SDC1, NOG, PLIN1
2	4.5	5	9	CXCR4, CCR5, CXCL9, CXCL16, IL33
3	2.667	4	4	IL21R, IL17 F, EGF, CRP

### Effects of piceatannol and epicatechin on proliferation, cell cycle progression, apoptosis and inflammatory response of RA-FLS

**Fig. 7:**
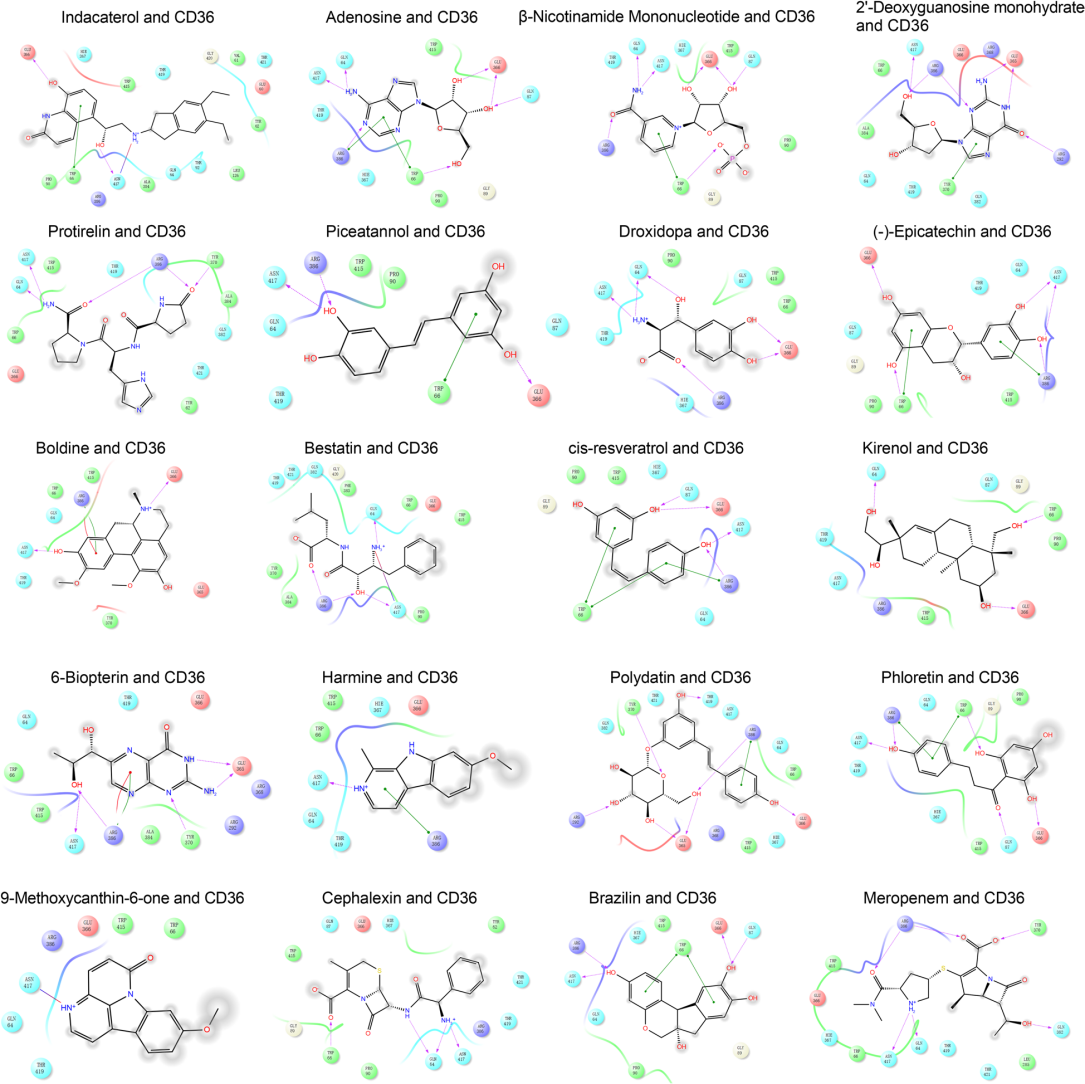
2D diagram of molecular docking patterns between CD36 protein and natural compounds

**Fig. 8:**
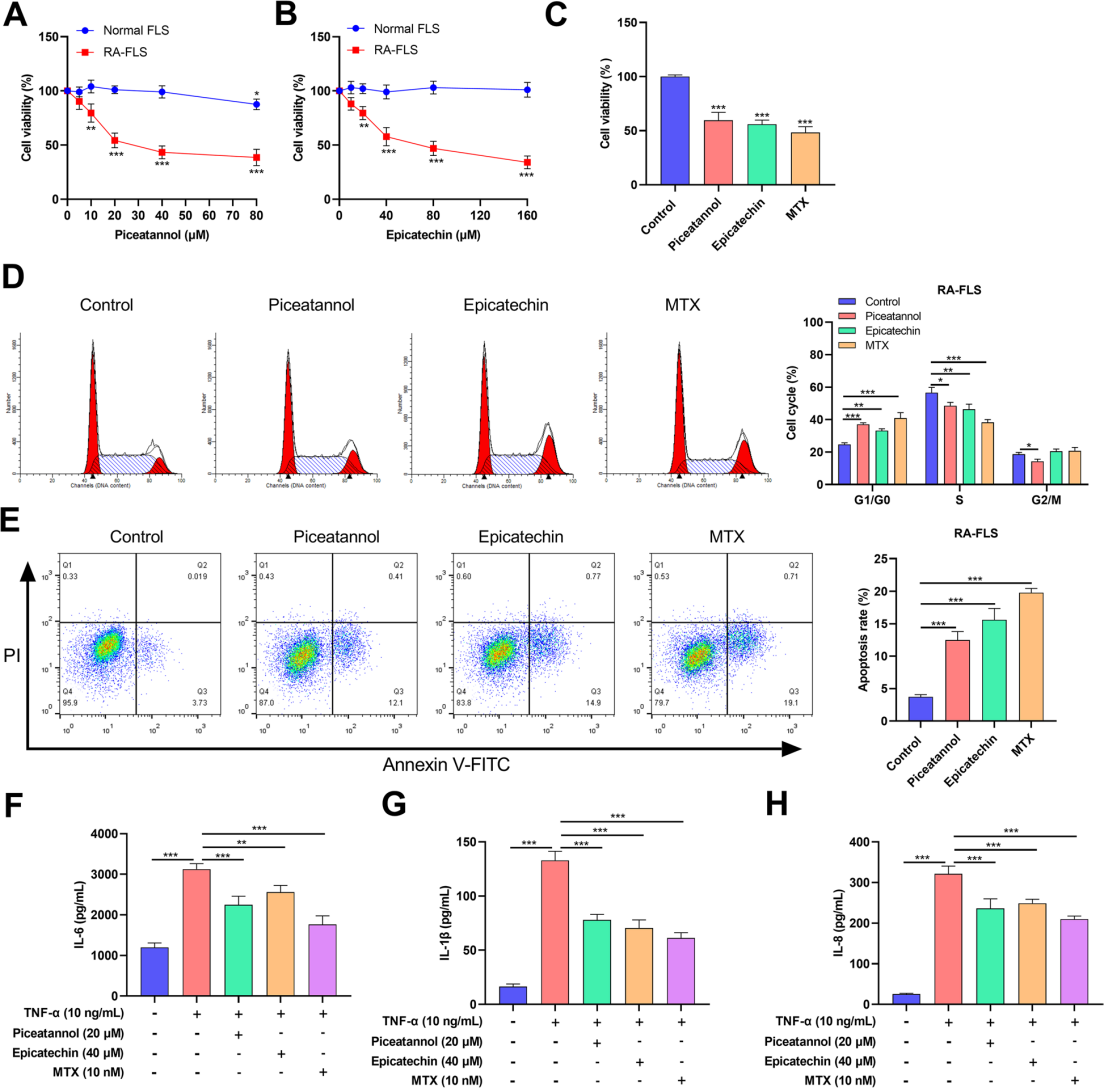
Effect of piceatannol or epicatechin on the phenotypes of RA-FLS. **A** and **B** The effects of different doses of piceatannol treatment (A) and epicatechin treatment (B) on the viability of RA-FLS and normal FLS were examined by CCK8 assay. The effects of 40 μM piceatannol and 160 μM epicatechin on the viability of RA-FLS were examined with CCK-8 assay, and methotrexate (MTX) (10 μM) was used as the positive control. **D** The effects of piceatannol treatment (E) and epicatechin treatment (F) on the cell cycle progression of RA-FLS were detected by flow cytometry. MTX was used as the positive control. **E** The effects of piceatannol treatment (G) and epicatechin treatment (H) on the apoptosis of RA-FLS were detected by flow cytometry. MTX was used as the positive control. **F**–**H**. The contents of IL-6 (F), IL-1β (G) and IL-8 (H) in the supernatant of RA-FLS cells induced by TNF-α (10 ng/ml) were detected by ELISA. MTX was used as the positive control. **P < 0.01, ***P < 0.001

In order to identify potentially drug for RA treatment, virtual screening was applied to screen the natural monomers probably targeting CD36. The top 20 compounds with high docking score are shown (Table 2, Fig. 7), including protirelin and epicatechin, and all of the docking scores were less than −5 kcal/mol. CCK-8 assay results showed that piceatannol treatment and epicatechin treatment significantly inhibited the proliferation of RA-FLS, respectively (*P* < 0.05, compared with the control group); however, the cytotoxicity of piceatannol (≤ 40 μM) and epicatechin (≤ 160 μM) were minor for normal FLS (*P* > 0.05, compared with the control group) (Fig. 8A&B). CCK-8 assay also showed piceatannol treatment (40 μM) and epicatechin treatment (160 μM) had similar cytotoxicity on the viability of RA-FLS with the positive control MTX (10 μM) (*P* < 0.05, compared with the blank group) (Fig. 8C). Flow cytometry analysis showed that piceatannol treatment and epicatechin treatment resulted in the increase of G0/G1 and G2/M phase cells, and the decrease of S phase cells (*P* < 0.05, compared with the control group) (Fig. 8D), and additionally, piceatannol treatment and epicatechin treatment also promoted the apoptosis of RA-FLS cells (*P* < 0.05, compared with the control group) (Fig. 8E). In addition, ELISA showed significantly increased levels of IL-6, IL-1β, and IL-8 in RA-FLS after TNF-α stimulation, while piceatannol treatment and epicatechin treatment attenuated this effect (*P* < 0.05, compared with the control group) (Fig. 8F-H). These results suggest that targeting CD36 by piceatannol or epicatechin can inhibit RA-FLS proliferation and TNF-α-induced inflammatory response, and promote cell cycle arrest and apoptosis.

**Table 2:** Active small molecules targeting CD36 protein based on virtual screening

No	Compound name	CAS	Docking score	Chemical structure
1	Indacaterol	312,753–06-3	−7.027	
2	Adenosine	58–61-7	−6.604	
3	β-Nicotinamide Mononucleotide	1094–61-7	−6.237	
4	2'-Deoxyguanosine monohydrate	961–07–9	−5.904	
5	Protirelin	24,305–27-9	−5.795	
6	Piceatannol	10,083–24-6	−5.749	
7	Droxidopa	23,651–95-8	−5.729	
8	(-)-Epicatechin	490–46-0	−5.725	
9	Boldine	476–70-0	−5.665	
10	Bestatin	58,970–76-6	−5.641	
11	cis-resveratrol	61,434–67-1	−5.628	
12	Kirenol	52,659–56-0	−5.616	
13	6-Biopterin	22,150–76-1	−5.579	
14	Harmine	442–51-3	−5.551	
15	Polydatin	65,914–17-2	−5.549	
16	Phloretin	60–82-2	−5.532	
17	9-Methoxycanthin-6-one	74,991–91-6	−5.485	
18	Cephalexin	15,686–71-2	−5.476	
19	Brazilin	474–07-7	−5.373	
20	Meropenem	96,036–03-2	−5.326

## Discussion

RA is a systemic autoimmune disease, its pathogenesis is related to synovial lesions, accompanied by synovial hyperplasia and inflammation. Inhibition of inflammatory response is considered to be an effective treatment strategy for RA, such as targeting TNF, IL-6, CD86/80 or janus kinase (JNK), and is potential to be an effective supplement for disease-modifying antirheumatic drugs (DMARDs) [15–17]. Given the prominent role of the inflammatory response in RA, it is critical to understand the exact mechanism of synovial inflammation. This study attempted to identify and validate hub regulators/therapeutic targets in RA synovium through bioinformatics analysis, and explore potential natural drugs. Based on two transcriptome datasets (GSE77298 and GSE206848), 87 DE-IRGs were obtained in the present work. Functional enrichment analysis suggested that these DE-IRGs were probably associated with cell chemotaxis, inflammation regulation, signaling receptor activator activity, functions of cytokines, etc. Abnormal cell chemotaxis is a key link in the persistence of inflammation and joint destruction in RA. Abnormal expression of chemokines and chemokine receptors can cause abnormal recruitment of various inflammatory cells, such as dendritic cell chemotaxis mediated by CCR5 and CD4 + CD161 + T cell chemotaxis mediated by CD147 [18, 19], etc. These inflammatory cells secrete a large amount of inflammatory cytokines in the lesion, aggravating joint injury. Our findings are consistent with these previous reports.

Further, combined with WGCNA and PPI network analysis, 3 hub genes were screened out, namely CD36, PLIN1 and LPL. It is worth noting that the expression of CD36 in RA was significantly higher than that in NC group through the validation of a external dataset GSE93272, and it had a good diagnostic ability of RA. It has been reported that CD36 is upregulated in the plasma of RA patients [20], which is consistent with the results of this study. Additionally, mRNA and protein levels of CD36 in RA-FLS were significantly up-regulated compared to normal FLS cells, which further suggested the regulatory function of CD36 in RA pathogenesis. CD36 is a highly glycosylated single-chain transmembrane protein that is expressed on the surface of various cell types, such as adipocytes, monocytes, macrophages, platelets, endothelial cells, etc. [21]. As a class B scavenger receptor, CD36 recognizes and binds to a variety of lipids, such as oxidized low-density lipoprotein (ox-LDL) and long-chain fatty acids, triggering a series of signaling pathways (such as NF-κB and toll-like receptor signaling pathways) that promote the production and release of inflammatory cytokines [22–26]. These inflammatory cytokines, such as TNF-α, IL-1β, IL-6 and IL-8, play a central role in the pathogenesis of RA. They not only exacerbate the inflammatory response, but also promote the abnormal proliferation of synovial cells and the destruction of articular cartilage [27–29]. Targeting TNF-α induced inflammation and proliferation may be an important strategy for the prevention and treatment of RA [30]. FLS is a major cellular component that maintains synovial homeostasis and plays an important role in synovial hyperplasia and inflammation of RA [30]. It has been reported that increased proliferation and inhibition of apoptosis of FLS greatly promote the occurrence and development of RA [31]. In this study, we demonstrated for the first time that inhibiting CD36 can inhibit proliferation and promote cell cycle arrest and apoptosis of RA-FLS. These findings suggest that CD36 is closely related to the progression of RA and may be a new therapeutic target, and its detailed mechanism in promoting RA progression deserves further investigation in the future.

Structure-Based Virtual Screening (SBVS) is an important method in computer-aided drug design, which is mainly used to select potentially active compounds from massive compound libraries [32]. In this study, based on virtual screening and molecular docking analysis, we identified a series of natural compounds that reliably bind with the active pocket of CD36, including piceatannol, epicatechin, cis-resveratrol, harmine, polydatin, phloretin, 9-Methoxycanthin-6-one and Brazilin. Piceatannol has been reported to inhibit RA-FLS inflammation and promote apoptosis by inhibiting NF-κB and MAPK signaling pathways [33]. (-) -epicatechin belongs to the polyphenolic flavonoid, which has been proven to be capable of scavenging free radicals and inhibiting lipid peroxidation [34]. cis-resveratrol is a non-flavonoid polyphenol compound. Studies have shown that resveratrol can promote apoptosis and G2/M cell cycle arrest of RA-FLS by regulating autophagy and the AKT-p53 axis [35]. Harmine is a β-caroline alkaloid, which can be extracted from plants such as Peganum harmala and has neuroprotective, anti-diabetic, anti-angiogenic, antiviral and anti-inflammatory activities [36]. Polydatin is a natural and effective derivative of stilbene polyphenols and resveratrol, which has anti-inflammatory, antioxidant and anti-apoptotic activities and has been proven to play a key role in rheumatoid diseases [37, 38]. As a flavonoid, Phloretin can inhibit the progression of RA by reducing the production of pro-inflammatory cytokines (TNF-α, IL-6, IL-1β and IL-17) [39]. 9-Methoxycanthin-6-one is a coumarin-like compound with antitumor activity [40]. Brazilin is a natural isoflavone-like red pigment with pro-apoptotic, anti-inflammatory and anti-proliferative potential. It has been reported that Brazilin can inhibit the inflammatory response of RA-FLS by enhancing autophagy by regulating the NF-κB pathway [41]. Our findings suggest that targeting CD36 will help develop new RA treatment strategies. As expected, in the present work, in vitro models showed that piceatannol or epicatechin can inhibit proliferation and TNF-α-induced inflammatory response of RA-FLS, and induced cell cycle arrest and apoptosis, validating the promising roles of piceatannol and epicatechin in RA treatment. Of course, the biological functions of picetannol and epicatechin in ameliorating RA awaits further validation with in vivo models in the future, and the roles of the other identified natural drugs in RA treatment should be explored in the following work.

There are certain limitations of the present work. First of all, even though this work demonstrated that CD36 was highly expressed in RA-FLS, and the data in GSE93272 suggested that it was up-regulated in the peripheral blood samples of RA patients, its diagnostic value should be further validated with a larger cohort with more patients from different medical centers, especially the AUC in the present dataset was less than 0.7, which was not suitable for clinical application. Additionally, even though the present work implied that CD36 was a potential drug target in RA treatment, considering there is no widely approved CD36 inhibitor, the no positive controls were set in virtual screening process and functional experiments. In the following work, monoclonal antibody of CD36 may be used to investigate the therapeutic value of targeting CD36 in RA treatment, and evaluate the advantages/disadvantages of piceatannol and epicatechin.

In conclusion, CD36 is a novel biomarker and therapeutic target for RA. Picetannol and epicatechin probably target CD36 to ameliorate RA. Our findings will contribute to a better understanding of the pathogenesis of RA and the development of new treatment strategies for RA.

## Data Availability

The data used to support the findings of this study are available from the corresponding author upon request.
